# A novel scoring system integrating molecular abnormalities with IPSS-R can improve the risk stratification in patients with MDS

**DOI:** 10.1186/s12885-021-07864-y

**Published:** 2021-02-06

**Authors:** Siyu Gu, Jingya Xia, Yulu Tian, Jie Zi, Zheng Ge

**Affiliations:** Department of Hematology, Zhongda Hospital, School of Medicine, Southeast University, Institute of Hematology Southeast University, Nanjing, 210009 China

**Keywords:** Myelodysplastic syndrome, Molecular abnormalities, Prognostic factors, Real-world study

## Abstract

**Background:**

The treatment strategies for Myelodysplastic Syndromes (MDS) are usually based on the risk stratification system. However, few risk signatures which integrate the revised international prognostic scoring system (IPSS-R) with gene mutations can be easily applied in the real world.

**Methods:**

The training cohort of 63 MDS patients was conducted at Zhongda Hospital of Southeast University from January 2013 to April 2020. The validation cohort of 141 MDS patients was obtained from GSE129828. The mutation scoring system was based on the number of mutations and a unique favorable prognostic factor, which is *SF3B1* mutation. Univariate Cox, multivariate Cox, and LASSO regression analyses were used to determine the significant factors that influenced the overall survival. The receiver operating characteristic curve (ROC) was used to evaluate the efficiency of the prognostic model.

**Results:**

A novel risk scoring system we named “mutation combined with revised international prognostic scoring system (MIPSS-R)” was developed based on the results derived from multivariate analysis which assigned points to the IPSS-R and the mutation scores according to their relative statistical weight. Based on the quintile of the new scores, patients were divided into five risk levels. The Kaplan-Meier curves showed the superiority of MIPSS-R in separating patients from different groups, comparing with IPSS-R both in the training cohort (*p* = 1.71e-08 vs. *p* = 1.363e-04) and validation cohort (*p* = 1.788e-04 vs. *p* = 2.757e-03). The area under the ROC of MIPSS-R was 0.79 in the training cohort and 0.62 in the validation cohort. The retrospective analysis of our house patients showed that the risk levels of 57.41% of patients would adjust according to MIPSS-R. After changing risk levels, 38.71% of patients would benefit from treatment strategies that MIPSS-R recommends.

**Conclusion:**

A mutation scoring system was conducted based on the number of mutations and a unique favorable prognostic factor. MIPSS-R, the novel integral risk stratification system was developed by integrating IPSS-R and the mutation scores, which is more effective on prognosis and treatment guidance for MDS patients.

**Supplementary Information:**

The online version contains supplementary material available at 10.1186/s12885-021-07864-y.

## Background

Myelodysplastic syndromes (MDS) are a group of clonal hematopoietic stem cell disorders characterized by ineffective and dysplastic hematopoiesis that causes cytopenia, which are also likely to progress to the development of acute myeloid leukemia (AML) [1, 2]. MDS is predominantly diagnosed among older adults, and more than half of the patients exceed the age of 75 [3, 4]. The treatment strategies are usually based on the risk stratifications like the revised International Prognostic Scoring System (IPSS-R), which consists with bone marrow (BM) cytogenetics, blast percentage, and peripheral blood (PB) cytopenia [5]. However, with the rapid development of high through-put technology like next-generation sequence (NGS), multiple mutations have been revealed as significant factors in MDS [6], and approximately 90% of MDS patients have at least one mutation [7]. One large-scale genomic research showed that *TET2*, *SF3B1*, *ASXL1*, *SRSF2*, *DNMT3A*, and *RUNX1* mutations appeared in more than 10% of MDS cases, and many mutations were correlated to higher risk groups or high blast counts [6]. According to National Comprehensive Cancer Network (NCCN) guidelines, epigenetic mutations such as *TET2*, *DNMT3A*, *ASXL1*, *IDH1/2,* and *EZH2* commonly occur in MDS; Splicing factor-related mutations such as *SF3B1*, *SRSF2*, *U2AF1* and *ZRSR2* are not specific mutations of MDS but occur more frequently in MDS than in other myeloid tumors. *SF3B1* mutation predicts a good prognosis; *SRSF2*, *RUNX1*, *U2AF1*, *ASXL1,* and *TP53* mutation predict high risks of progressing to AML [8]. It is well known that mutations have the prognostic effect in MDS [9, 10], but a perfect scoring system based on mutation or combined with IPSS-R has not yet appeared.

Based on these concepts, we would like to build a novel prognostic system that integrated the mutations with IPSS-R. To address these issues and to expand the knowledge about predictive factors, the data of 63 patients from our clinical center was utilized as a training cohort, and the data of 141 patients from GSE129828 was utilized as a validation cohort. This research aims to provide physicians with practical information, support them in choosing the best treatment plan, and provide consultations for patients.

## Methods

### Patient cohorts

A total of 63 de novo MDS patients were collected in the department of hematology, Zhongda Hospital from January 2013 to April 2020, which were conducted as the training cohort. The diagnostic criteria and subclassified standards were referenced to the World Health Organization (WHO) in 2008 [11]. All samples were collected with patient consent under protocols approved by institutional review boards and by the Declaration of Helsinki. The prognostic risk stratifications of all patients were according to the IPSS-R prognosis integral systems [5, 12].

Meanwhile, A total of 141 patients with treatment-naive MDS from dataset GSE129828 [13] were composed to a validation cohort, which was similar to the training cohort and was obtained from the Gene Expression Omnibus (https://www.ncbi.nlm.nih.gov/geo/). Clinical data to determine the IPSS-R scores, mutations and French-American-British (FAB) classification were available at the time of sample collection.

### Cytogenetic and molecular biology determination

All patients in the training cohort submitted BM aspirates at the time of admission. Cytogenetic analysis was conducted by conventional G-banding technology and fluorescence in situ hybridization (FISH). Each sample with three or more abnormal genetic characteristic BM cells was considered a sample with abnormal clones after analyzing at least 20 metaphases. Taking the FISH examination, each probe analyzed at least 200 cells. When the proportion of abnormal signal cells of a sample exceed the threshold, the sample considered with cytogenetic abnormality. By using DNA extracted from each aspirate, the mutational analysis was taken with an amplicon-based, NGS panel targeting the entire coding regions of 31 genes frequently mutated in MDS (supplement Table 1). Only mutations that have been previously reported to be pathogenic either in the Catalogue of Somatic Mutations in Cancer (COSMIC) ID or other databases or in the literature were considered in the present study.

### Mutation risk stratification and MIPSS-R

The mutation risk stratification was constructed by the number of mutated genes and only one favorable prognostic mutated gene. Patients with no mutant or with only *SF3B1* mutation classified into the low-risk; with one mutant except *SF3B1* classified into intermediate-1-risk; with two to four mutants classified into intermediate-2-risk; with five or more mutants classified into high-risk. Patients in low, intermediate-1, intermediate-2, and high risk were assigned 0, 1, 2, and 3 points, respectively. The mutation combined with revised international prognostic scoring system (MIPSS-R) was developed based on the results derived from multivariate analysis which assigned points to the IPSS-R and the mutation scores according to their relative statistical weight. Based on the quintile of the MIPSS-R scores, patients were divided into very low-, low-, intermediate-, high-, and very high-risk groups, respectively.

### Statistical analysis

The overall survival (OS) was defined as the time in days from the date of MDS diagnosis to the date of last follow-up or death, regardless of causes. The univariate, multivariate Cox and Least absolute shrinkage and selection operator (LASSO) regression models, receiver operating characteristic curve (ROC) analyses, and Kaplan-Meier (K-M) survival curve with Log Rank analysis were performed using R studio (version 3.6.3). The univariate, multivariate Cox regression and K-M survival analyses were performed with the package of “survival” in R. The LASSO regression analysis was performed with R package of “glmnet” in R. The prediction ability of the model was assessed by the area under the curve (AUC) of ROC with the package of “survivalROC” in R. Quantitative data were exhibited as the mean ± standard deviation (SD). Mann-Whitney U test and Fisher exact test analyzed continuous variable and categorical variables respectively by using SPSS 26.0 software. All statistical tests were bilateral, with a *p*-value < 0.05 being statistically significant.

## Results

### Baseline characteristics

The follow-up deadline of the training cohort was April 20, 2020. 9 out of 63 patients were removed due to losing follow-up. In the validation cohort, 33 out of 141 patients were removed from the present study since lack of survival data. Finally, data of a total of 162 patients were analyzed, 54 patients in the training cohort and 108 patients in the validation cohort. The clinical characteristics for each patient were summarized in Table 1. The baseline characters including the age, gender, BM blasts proportion, and mutation risk stratifications had no differences between the two cohorts. Most of the patients were low-risk ones according to the IPSS-R category, 38.9% of patients in the training cohort, and 28.7% in the validation cohort (*p* = 0.015). The most common subtypes in the training cohort were multilineage dysplasia (MLD, 42.6%) based on 2008 WHO classification, and in the validation cohort were refractory anemia with excess of blast (RAEB, 35.2%) based on FAB classification.

**Table 1:** Baseline patient characteristics between the training cohort and validation cohort

Characteristics	Total		Training		Validation		*p*
	No.	%	No.	%	No.	%	
Total	162		54		108		
Age, years							0.075
Median	71		70		72		
Range	24–91		24–88		48–91		
Male sex	112	69.10%	33	61.10%	79	73.10%	0.149
ANC, 10^9^/L							0.007
Median	1.4		1.18		1.6		
Range	0.07–28.04		0.07–6.33		0.16–28.04		
Hemoglobin, g/dL							< 0.0001
Median	9.2		7.05		9.7		
Range	3.3–15.2		3.30–13.4		5.80–15.20		
Platelets, 10^9^/L							< 0.0001
Median	81.5		53		101		
Range	2–987		2–310		6–987		
Blasts %							0.655
Median	1		0.8		1		
Range	0–28		0–18.8		0–28		
IPSS-R Category							0.015
Very low	22	13.6	1	1.9	21	19.4	
Low	52	32.1	21	38.9	32	28.7	
Intermediate	31	19.1	13	24.1	18	16.7	
High	35	21.6	11	20.4	24	22.2	
Very high	22	13.6	8	14.8	14	13	
Mutation risk stratification							0.156
Low	30	18.5	11	20.4	19	17.6	
Intermediate-1	39	24.1	13	24.1	26	24.1	
Intermediate-2	86	53.1	25	46.3	61	56.5	
High	7	4.3	5	9.3	2	1.9	
MIPSSR							0.956
Very low	31	19.1	10	18.5	21	19.4	
Low	29	17.9	11	20.4	18	16.7	
Intermediate	33	20.4	10	18.5	23	21.3	
High	36	22.2	11	20.4	25	23.1	
Very high	33	20.4	12	22.2	21	19.4	
2008 WHO classification							NA
MDS-SLD			5	9.3			
MDS-MLD			23	42.6			
MDS-RS			7	13.0			
MDS-EB1			8	14.8			
MDS-EB2			8	14.8			
MDS-U			3	5.6			
FAB classification							NA
RA					36	33.3	
RARS					20	18.5	
RAEB					38	35.2	
RAEB-T					6	5.6	
RCUD					2	1.9	
RCMD					2	1.9	
MDS-U					1	0.9	
CMML					3	2.8	

In terms of the mutation risk stratification, most of the patients were in the intermediate-2 risk group, both in the training cohort (46.3%) and validation cohort (56.5%, Table 1). The most common mutations were *ASXL1*, *TET2*, *TP53*, *SRSF2*, and *SF3B1*, accounting for 31.5, 27.8, 18.5, 14.8, and 14.8%, respectively, in the training cohort; meanwhile, the most common mutations in the validation cohort were *TET2*, *ASXL1*, *SF3B1*, *RUNX1*, and *SRSF2*, making up 31.5, 27.8, 25, 17.6, and 16.7%, respectively. A total of 20 same mutated genes were detected in both two cohorts, and there was no differences between cohorts (Supplement Table 2). In terms of abnormal karyotypes, 5q- was the most common one both in the training cohort (20.4%) and the validation cohort (9.3%). Complex karyotype was defined as more than or equal to three abnormal karyotypes, constituting 9.3% in the training cohort and 8.3% in the validation cohort. The expression of karyotype was similar between the two cohorts except for the 20q- (*p* = 0.007, Supplement Table 3).

### Survival analysis

**Fig. 1:**
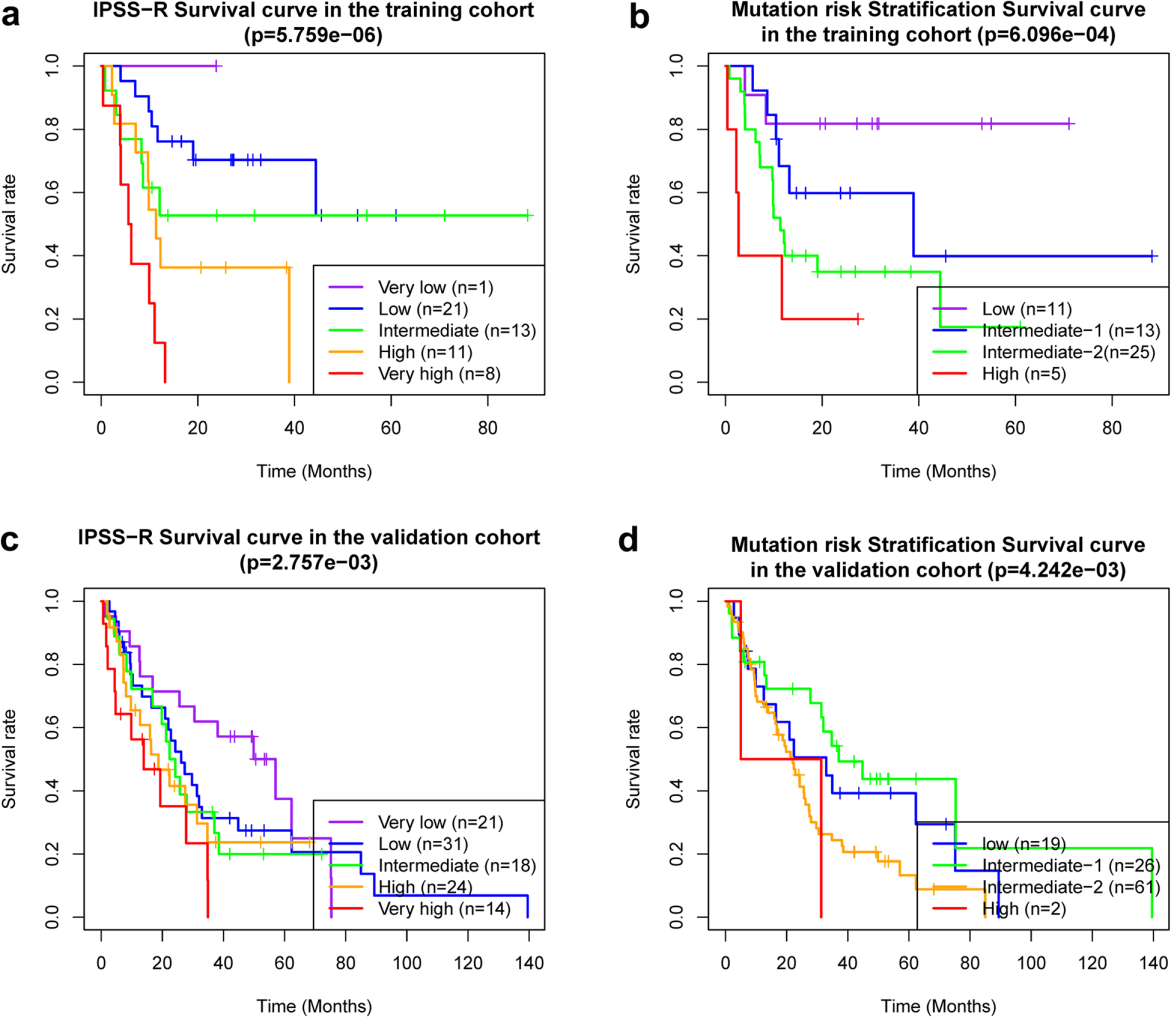
**a**, **b** The Kaplan-Meier curve of patients from the training cohort; **c**, **d** The Kaplan-Meier curve of patients from the validation cohort. **a**, **c** The Kaplan-Meier curves of patients in different IPSS-R risk stratifications. **b**, **d** The Kaplan-Meier curves of patients in different mutation risk stratifications

For the training cohort, with a median follow up of 13.5 months (range, 0.39–88.24 months), the median OS per IPSS-R scoring system was > 60, > 60, > 60, 11.34, and 5.92 months for very low-, low-, intermediate-, high-, and very high-risk, respectively, *p* = 5.759e-06 (Fig. 1 a). The median OS per mutation risk stratification was > 60, 38.9, 11.3, and 2.7 months for low-, intermediate-1-, intermediate-2-, and high-risk, respectively, *p* = 6.096e-04 (Fig. 1 b). For the validation cohort, with a median follow up of 22.2 months (range, 0.66–139.51 months), the median OS per IPSS-R scoring system was 25.59, 20.93, 16.8, 9.73, and 4.7 months for very low-, low-, intermediate-, high-, and very high-risk, respectively, *p* = 2.757e-03(Fig. 1 c). The median OS per mutation risk stratification was 33, 37, 22, and 18.2 months for low-, intermediate-1-, intermediate-2-, and high-risk, respectively, *p* = 4.242e-03 (Fig. 1 d).

To identify all independent factors for OS, we next performed univariate Cox proportional hazards regression analysis and LASSO regression analysis in the training cohort. Univariate analysis demonstrated that age, *TP53* mutation, mutation risk stratifications, IPSS-R, progression to AML, + 8, − 7/7q-, and complex karyotype were the prognostic factors (supplement Fig. 1 a). LASSO regression analysis was performed to select factors, and − 7/7q-, IPSS-R, and mutation risk stratification were retained according to the optimal lambda value [log(lambda.min) = − 1.64, supplement Fig. 1 b, c]. Next, in multivariate analysis, we confirmed the IPSS-R (*p* <  0.01) and mutation risk stratification (*p* <  0.001) as significant predictors for OS (supplement Fig. 1 d).

### Incorporating mutation risk stratification into IPSS-R

**Fig. 2:**
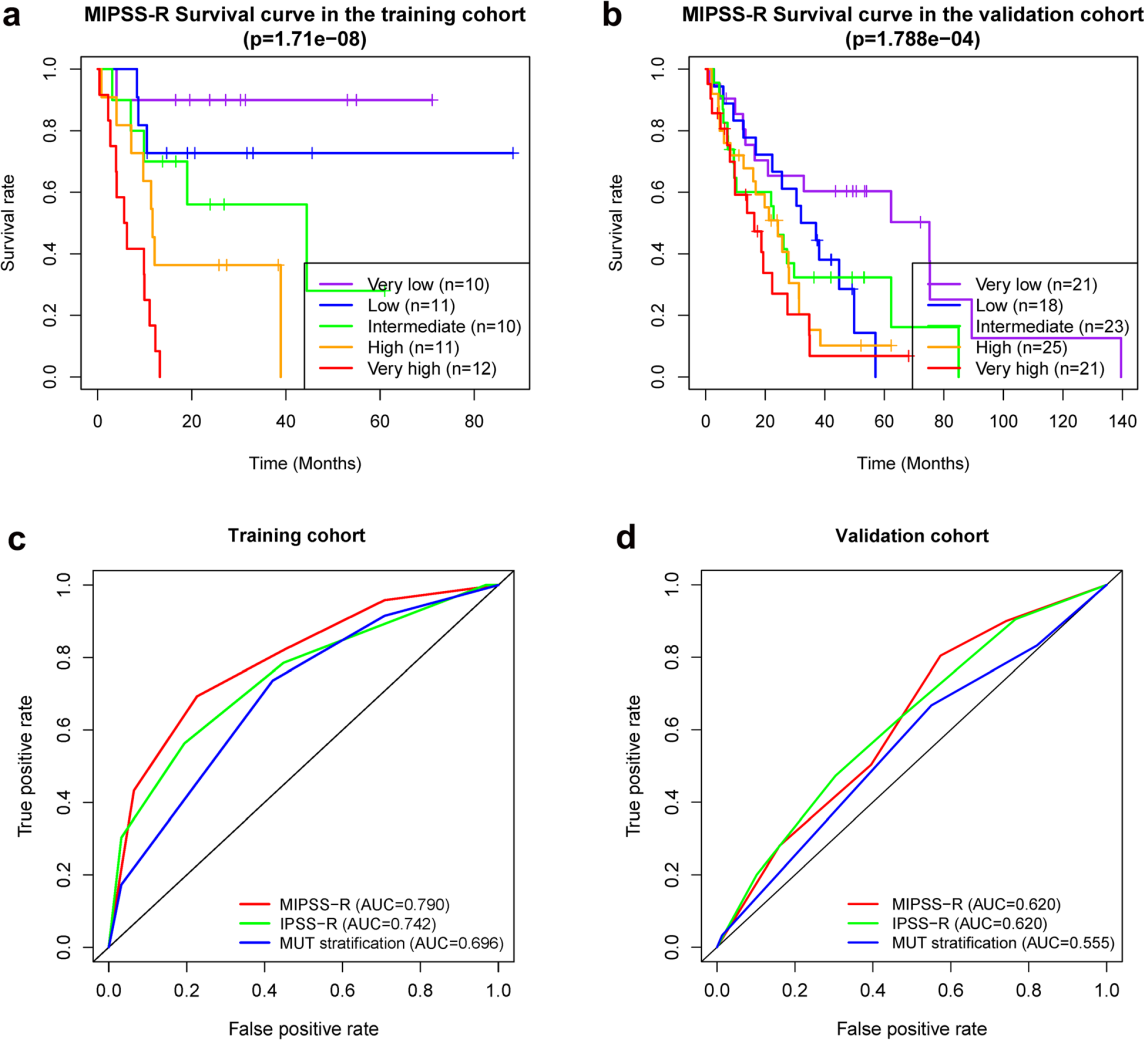
**a** The Kaplan-Meier curves of patients in different MIPSS-R risk stratifications and **c** the receiver operating characteristic curves of different risk stratifications in the training cohort; **b** The Kaplan-Meier curves of patients in different MIPSS-R risk stratifications and **d** the receiver operating characteristic curves of different risk stratifications the validation cohort

In the next step, we aimed for the development of a practical risk score based on the results derived from multivariate analysis. A novel risk scoring system, we named MIPSS-R was developed based on a linear combination of the mutation risk stratification score and the IPSS-R score multiplied by regression coefficients obtained from the multivariate analysis: MIPSS-R score = mutation score × 1.047 + IPSS-R × 0.641. Based on the quintile of the MIPSS-R scores (ranged from 1.28 to 8.59), patients were divided into very low- (1.28–2.24), low- (2.33–3.93), intermediate- (4.02–4.34), high- (4.57–5.30), and very high-risk (5.62–8.59) with the median OS of > 60, > 60, 44.41, 11.68, and 5.92 months respectively (Fig. 2 a, *p* = 1.71e-08). We then calculated the MIPSS-R scores of the patients in the validation cohort. The OS was significantly different between groups (*p* = 1.788e-04) with the median OS of 75.1, 34.5, 24.2, 24.2, and 16.3 months in very low-, low-, intermediate-, high-, and very high-risk, respectively (Fig. 2 b). Meanwhile, the AUC value of the MIPSS-R was 0.790, which was higher than IPSS-R (0.731) and mutation scoring system (0.672) alone in the training cohort (Fig. 2 c). MIPSS-R in the validation cohort had an equal AUC value to the IPSS-R (0.620), but higher than the mutation scoring system (0.555, Fig. 2 d).

### The clinical significance of the MIPSS-R

To highlight the clinical significance of the MIPSS-R, we compared the risk stratifications changes in the training cohort (supplement Table 4). 27.78% (15/54) patients had a decreased risk, and 29.63% (16/54) patients had an elevated risk. Patients #49 and #58 were classified as intermediate-risk and low-risk based on IPSS-R and received demethylation therapy but died shortly owing to toxicity thereafter. These two patients would benefit from supportive care or other non-intensive therapy based on MIPSS-R guidance. Moreover, patient #4, #18, and #41 were classified into low-risk and patients #36 and #44 were intermediate-risk based on IPSS-R, who eventually died as they only received supportive care. These patients might receive more intensive therapy and would expect a better prognosis based on MIPSS-R suggestion. Further, patients #5, #32, #34, #48, and #53 were grouped into low-risk based on IPSS-R but received demethylation therapy or more intensive therapy instead of supportive therapy, who achieved better outcomes. Notably, patients #4, #40, #53, and #61 with elevated risk eventually developed to AML, which indicated the possibility of early identification of AML progression by MIPSS-R.

## Discussion

MDS are a group of highly heterogeneous diseases which are commonly occurred in the elderly population, characterized by pancytopenia and a high risk of progressing to AML. Various factors including the BM blasts, pancytopenia, cytogenetic characteristics, and genetic mutations affect the prognosis of the disease [14]. The gold criteria for assessing conditions of MDS patients is IPSS-R which is the latest version of IPSS revised by the MDS Prognosis International Working Group in 2012 [5]. However, independent prognostic elements such as red blood cell transfusion dependence, genetic mutations are not included in the scoring system, especially the gene mutations that help to the accurate assessment [15]. The advance of modern technology has improved the genome-wide analysis of genetic mutations in MDS [6, 16]. Although the evolution of molecular technology has introduced new challenges, it is also leading to novel recognition of accurate diagnosis and therapy.

One large scale molecular research analyzed 994 MDS patients and revealed the genomic landscape of the disease [6]. The most frequently mutated genes were *TET2*, *SF3B1*, *ASXL1*, *SRSF2*, *DNMT3A,* and *RUNX1*, that all accounted for more than 10% in these patients. Another whole-exome sequencing study of 699 patients revealed distinct patterns of clonal evolution in MDS [17]. The data showed that MDS patients with *SF3B1* mutations were enriched in the low-risk group, but patients with *GATA2*, *NRAS*, *KRAS*, *IDH2*, *TP53*, *RUNX1*, *STAG2*, *ASXL1*, *ZRSR2*, and *TET2* mutations were enriched in the high-risk group. Patients with *FLT3*, *PTPN11*, *WT1*, *IDH1*, *IDH2*, *NPM1*, and *NRAS* mutations were significantly correlated to the AML progression. Meanwhile, they also found that most of the patients have unique mutated patterns, leading to a great deal of heterogeneity [6, 17].

In the past few years, some studies have integrated mutations with IPSS-R to improve the prognostic values for MDS patients. One of the researches by Bejar et al. described a mutation landscape of 439 MDS patients and screened out five mutations, *TP53*, *EZH2*, *ETV6*, *RUNX1*, and *ASXL1*, that could predict the poor overall survival of MDS patients independently [18]. Haferlach et al. utilized the predictors including age, gender, IPSS-R, and 14 mutations genes, building a novel prognostic model (model-1) and separating patients into four risk groups, which showed significantly different 3-year survival rate of 95.2, 69.3, 32.8, and 5.3%, respectively [6]. Comparing with another model built by the 14 mutations alone (Model-2), and with IPSS-R, model-1 was more superior. Nazha et al. incorporated mutated *EZH2*, *SF3B1*, and *TP53* with IPSS-R and improved the predictive ability in MDS [19]. Notably, MDS patients enrolled in the study were serial samples with different time points during their disease courses. The new model classified patients into 4 risk groups with a median OS of 37.4, 23.2, 19.9, and 12.2 months, respectively. The new model also had a better C-index than IPSS-R. The results of the paired samples also confirmed the new model had a dynamic prognostic potential. Hou et al. built an integrated risk-stratification model consisting with the age, IPSS-R, and 5 mutations (*CBL*, *IDH2*, *DNMT3A*, *ASXL1*, and *TP53*) [20], diving patients into four risk groups, and the median OS of each group was 250.7, 38.4, 17, and 8.9 months respectively. They also showed that the new model could be well applied not only in the FAB-defined MDS patients but also those defined by WHO. Recently, Naqvi et al. also developed a new prognostic system incorporating 27-item Adult Comorbidity Evaluation (ACE-27) and *TP53* mutation with IPSS-R which improved outcome prediction in patients with MDS [7]. The C-index for the new model is 0.822, and the survival curves between risk groups of the new model were more well-separated than those of IPSS-R risk groups. The study also highlighted that clonal hematopoiesis of indeterminate potential (CHIP) associated mutations were associated with a higher frequency of prior history of cardiovascular events and poor prognosis in patients with MDS.

Certainly, the specific effects of some mutations are commonly accepted, besides the mutated genes aforementioned utilized in multivariable models, *DNMT3A*, *U2AF1*, *SRSF2*, *CBL*, *PRPF8*, *SETBP1*, and *KRAS* have also been reported the association with decreased OS [6, 18, 21–23]. Only mutated *SF3B1* is correlated to favorable outcome [6, 24]. Dr. Bejar. argue that the mutation patterns of MDS are diverse and no two are the same [25]. Due to the heterogeneities of mutations in MDS patients, it is difficult to utilize the prognostic model with specific mutants to assess patients with mutants that with uncertain prognostic values. Hence, we constructed a more simple-to-use mutation scoring system which contained only one favorable factor and the number of mutations, assigning 0 to 3 points to patients respectively. Then, the novel prognostic scoring system, MIPSS-R, was constructed by the linear combination of the IPSS-R and the mutation scoring system. Although MIPSS-R would lose some specific information about mutated genes, it overcomes the heterogeneities of mutation patterns of MDS patients. The prognostic value was also verified in the validation cohorts. Moreover, retrospective analysis of our house patients showed that more than half of the patients would adjust the risk stratification based on the MIPSS-R. Through a comprehensive analysis of the treatment strategies and outcomes of these patients, we found that part of the patients may obtain better prognosis under the guidance of MIPSS-R.

However, this study still has some limitations. First, our study was retrospective, so there may be some inherent biases. Secondly, the number of patients was limited and large-scale prospective researches are prospected. Third, the prognostic model needs to be verified in other cohorts, such as specific treatment strategies cohort and paired cohort.

## Conclusions

In summary, by integrating IPSS-R and gene mutations, MIPSS-R, a novel risk stratification system for MDS patients has been developed. This system is more effective in prognosis and will be helpful to reduce treatment-related deaths, to recognize MDS patients with high-risk or AML progression risk earlier.

## Supplementary Information

**Additional file 1.**

## Data Availability

Patients’ data that before December 2018 support the findings of this study are available on request from the Beijing Hyster Technology Co., Ltd. Patients’ data after December 2018 that support the findings of this study are available on request from the corresponding author. Data from GSE129828 was obtained from the Gene Expression Omnibus (https://www.ncbi.nlm.nih.gov/geo/).

## Abbreviations

MDS: Myelodysplastic Syndromes
IPSS-R: Revised International Prognostic Scoring System
ROC: The receiver operating characteristic curve
AML: Acute myeloid leukemia
BM: Bone marrow
PB: Peripheral blood
NGS: Next-generation sequence
NCCN: National Comprehensive Cancer Network
WHO: World Health Organization
FISH: Fluorescence in situ hybridization
COSMIC: Catalogue of Somatic Mutations in Cancer
OS: Overall survival
LASSO: Least absolute shrinkage and selection operator
K-M: Kaplan-Meier
AUC: Area under the curve
SD: Standard deviation
MDS-SLD: MDS with single lineage dysplasia
MDS-MLD: MDS with multilineage dysplasia
MDS-RS: MDS with ring sideroblasts
MDS-5q-: MDS with isolated del 5q
MDS-EB: MDS with excess blasts
MDS-U: MDS, unclassified
BM: Bone marrow
ANC: Absolute neutrophil count
Hb: Hemoglobin
PLT: Platelet
MIPSS-R: The mutation combined with revised international prognostic scoring system
