# Supplementary material for: A novel scoring system integrating molecular abnormalities with IPSS-R can improve the risk stratification in patients with MDS
Source: BMC Cancer. 2021 Feb 6;21:134. doi: 10.1186/s12885-021-07864-y (PMC7866647; doi:10.1186/s12885-021-07864-y)
Supplement: Supplementary file 1 — Additional file 1. [file 12885_2021_7864_MOESM1_ESM.zip › supplement filesR4.docx]

Supplement Table 1. The 31 common mutations’ panel in Myelodysplastic Syndromes.

| ASXL1 | JAK2 | RUNX1 |
| --- | --- | --- |
| BCOR | KRAS | SETBP1 |
| CBL | MPL | SF3B1 |
| CEBPA | NF1 | SRSF2 |
| DNMT3A | NPM1 | STAG2 |
| ETV6 | NRAS | TET2 |
| EZH2 | PHF6 | TP53 |
| FLT3 | PPM1D | U2AF1 |
| GATA2 | PTPN11 | WT1 |
| IDH1 | PRPF8 | ZRSR2 |
| IDH2 |  |  |

Supplement Table 2. The overlapped mutation genes in the training cohort and the validation cohort.

| mutations | total | | training | | validation | | *p* |
| --- | --- | --- | --- | --- | --- | --- | --- |
|  | No. | % | No. | % | No. | % |  |
| ASXL1 | 47 | 29.0 | 17 | 31.5 | 30 | 27.8 | 0.714 |
| TET2 | 49 | 30.2 | 15 | 27.8 | 34 | 31.5 | 0.718 |
| TP53 | 19 | 11.7 | 10 | 18.5 | 9 | 8.3 | 0.071 |
| SRSF2 | 26 | 16.0 | 8 | 14.8 | 18 | 16.7 | 0.824 |
| SF3B1 | 35 | 21.6 | 8 | 14.8 | 27 | 25.0 | 0.160 |
| DNMT3A | 19 | 11.7 | 6 | 11.1 | 13 | 12.0 | 1.000 |
| U2AF1 | 20 | 12.3 | 5 | 9.3 | 15 | 13.9 | 0.458 |
| RUNX1 | 24 | 14.8 | 5 | 9.3 | 19 | 17.6 | 0.240 |
| EZH2 | 13 | 8.0 | 4 | 7.4 | 9 | 8.3 | 1.000 |
| WT1 | 4 | 2.5 | 3 | 5.6 | 1 | 0.9 | 0.108 |
| NPM1 | 6 | 3.7 | 2 | 3.7 | 4 | 3.7 | 1.000 |
| NRAS | 7 | 4.3 | 2 | 3.7 | 5 | 4.6 | 1.000 |
| IDH2 | 4 | 2.5 | 2 | 3.7 | 2 | 1.9 | 0.601 |
| CEBPA | 2 | 1.2 | 2 | 3.7 | 0 | 0.0 | 0.110 |
| PHF6 | 4 | 2.5 | 2 | 3.7 | 2 | 1.9 | 0.601 |
| BCOR | 2 | 1.2 | 2 | 3.7 | 0 | 0.0 | 0.110 |
| NF1 | 5 | 3.1 | 2 | 3.7 | 3 | 2.8 | 1.000 |
| GATA2 | 2 | 1.2 | 2 | 3.7 | 0 | 0.0 | 0.110 |
| KRAS | 3 | 1.9 | 1 | 1.9 | 2 | 1.9 | 1.000 |
| MPL | 1 | 0.6 | 1 | 1.9 | 0 | 0.0 | 0.333 |

Supplement Table 3. The overlapped karyotypes between the training and validation cohorts.

| karyotype | total | | training | | validation | | *p* |
| --- | --- | --- | --- | --- | --- | --- | --- |
|  | No. | % | No. | % | No. | % |  |
| 5q- | 21 | 13 | 11 | 20.4 | 10 | 9.3 | 0.08 |
| isolate 5q- | 8 | 4.9 | 3 | 5.6 | 5 | 4.6 | 1 |
| 7q-/-7 | 15 | 9.3 | 7 | 13 | 8 | 7.4 | 0.262 |
| 20q- | 11 | 6.8 | 8 | 14.8 | 3 | 2.8 | 0.007 |
| +8 | 14 | 8.6 | 7 | 13 | 7 | 6.5 | 0.234 |
| -Y | 5 | 3.1 | 3 | 5.6 | 2 | 1.9 | 0.334 |
| Complex | 14 | 8.6 | 5 | 9.3 | 9 | 8.3 | 1 |

Supplement Table 4. The MDS patients with risk stratification adjusting after assessing by the novel risk stratification system.

| **ID** | **IPSS-R** | **Actual Treatment** | **Survival State (Progression)** | **Overall Survival (Years)** | **Novel Score** | **MIPSS-R** | **Recommended Treatment*** |  |
| --- | --- | --- | --- | --- | --- | --- | --- | --- |
| **Patients with decreased risk stratification** | | | | | | | | |
| #1 | intermediate risk | Supportive Care | Alive | 7.4 | 3.29 | low risk | Supportive Care and/or Hypomethylating Agents |  |
| #3 | intermediate risk | Hypomethylating Agents | Alive | 5.9 | 2.24 | low risk | Supportive Care and/or Hypomethylating Agents |  |
| #6 | intermediate risk | Supportive Care | Alive | 4.6 | 2.24 | low risk | Supportive Care and/or Hypomethylating Agents |  |
| #7 | low risk | Hypomethylating Agents +Thalidomide | Alive | 4.4 | 1.28 | very low risk | Supportive Care |  |
| #23 | intermediate risk | Hypomethylating Agents | Alive | 2.6 | 2.89 | low risk | Supportive Care and/or Hypomethylating Agents |  |
| #25 | low risk | Supportive Care | Alive | 2.6 | 1.28 | very low risk | Supportive Care |  |
| #26 | intermediate risk | Supportive Care | Dead | 0.7 | 3.93 | low risk | Supportive Care and/or Hypomethylating Agents |  |
| #28 | low risk | Hypomethylating Agents | Alive | 2.5 | 1.60 | low risk | Supportive Care and/or Hypomethylating Agents |  |
| #33 | low risk | Supportive Care | Alive | 2.3 | 1.92 | low risk | Supportive Care and/or Hypomethylating Agents |  |
| #47 | low risk | Supportive Care | Alive | 1.6 | 1.60 | very low risk | Supportive Care |  |
| #49 | intermediate risk | Hypomethylating Agents | Dead | 0.7 | 2.57 | low risk | Supportive Care and/or Hypomethylating Agents |  |
| #54 | low risk | Supportive Care | Alive | 1.4 | 1.69 | very low risk | Supportive Care |  |
| #58 | low risk | Hypomethylating Agents | Dead | 0.3 | 1.28 | very low risk | Supportive Care |  |
| #59 | intermediate risk | Supportive Care | Alive | 0.9 | 3.29 | low risk | Supportive Care and/or Hypomethylating Agents |  |
| #62 | high risk | Hypomethylating Agents +allo-HSCT | Alive | 1.7 | 3.85 | low risk | Supportive Care and/or Hypomethylating Agents |  |
| **Patients with elevated risk stratification** | | | | | | | | |
| #4 | low risk | Supportive Care | Dead (AML) | 3.7 | 4.02 | intermediate risk | Hypomethylating Agents and/or Intensive Chemotherapy |  |
| #5 | low risk | Hypomethylating Agents | Alive | 5.1 | 4.02 | intermediate risk | Hypomethylating Agents and/or Intensive Chemotherapy |  |
| #18 | low risk | Supportive Care | Dead | 1.6 | 4.02 | intermediate risk | Hypomethylating Agents and/or Intensive Chemotherapy |  |
| #24 | low risk | Hypomethylating Agents | Dead | 0.6 | 4.02 | intermediate risk | Hypomethylating Agents and/or Intensive Chemotherapy |  |
| #32 | low risk | Hypomethylating Agents | Alive | 2.3 | 4.74 | high risk | Intensive Chemotherapy |  |
| #34 | low risk | Hypomethylating Agents +HAG | Alive | 2.2 | 4.02 | intermediate risk | Hypomethylating Agents and/or Intensive Chemotherapy |  |
| #36 | intermediate risk | Supportive Care | Dead | 0.1 | 4.66 | high risk | Intensive Chemotherapy |  |
| #38 | intermediate risk | Hypomethylating Agents +HAG | Dead | 1.0 | 4.98 | high risk | Intensive Chemotherapy |  |
| #40 | high risk | Hypomethylating Agents +HAG | Dead (AML) | 0.8 | 5.94 | very high risk | Intensive Chemotherapy |  |
| #41 | low risk | Supportive Care | Dead | 1.0 | 4.74 | high risk | Intensive Chemotherapy |  |
| #44 | intermediate risk | Supportive Care | Dead | 0.3 | 4.98 | high risk | Intensive Chemotherapy |  |
| #50 | low risk | Hypomethylating Agents | Dead | 0.8 | 4.02 | intermediate risk | Hypomethylating Agents and/or Intensive Chemotherapy |  |
| #51 | low risk | Hypomethylating Agents | Dead | 1.0 | 5.62 | very high risk | Intensive Chemotherapy |  |
| #53 | low risk | Hypomethylating Agents +Ara-C | Alive (AML) | 1.4 | 4.02 | intermediate risk | Hypomethylating Agents and/or Intensive Chemotherapy |  |
| #60 | high risk | Hypomethylating Agents +Retinoids | Dead | 0.2 | 6.67 | very high risk | Intensive Chemotherapy |  |
| #61 | high risk | Hypomethylating Agents +HAG | Dead (AML) | 0.2 | 6.67 | very high risk | Intensive Chemotherapy |  |

Abbreviation: Ara-C, cytarabine; HAG, homoharringtonine, Ara-C, Granulocyte-colony stimulating factor; AML, acute myeloid leukemia; * Other factors affecting the treatment selection, such as the patient's age and physical condition were not considered.

Supplement figure: (a) Forest graph of univariate Cox regression analysis; (b) Log(Lambda) value of clinical factors in LASSO model; (c) The most proper log(Lambda) value in LASSO model; (d) Forest graph of multivariate Cox regression analysis.
