# Supplementary figures and images for: A novel scoring system integrating molecular abnormalities with IPSS-R can improve the risk stratification in patients with MDS
Source: BMC Cancer. 2021 Feb 6;21:134. doi: 10.1186/s12885-021-07864-y (PMC7866647; doi:10.1186/s12885-021-07864-y)

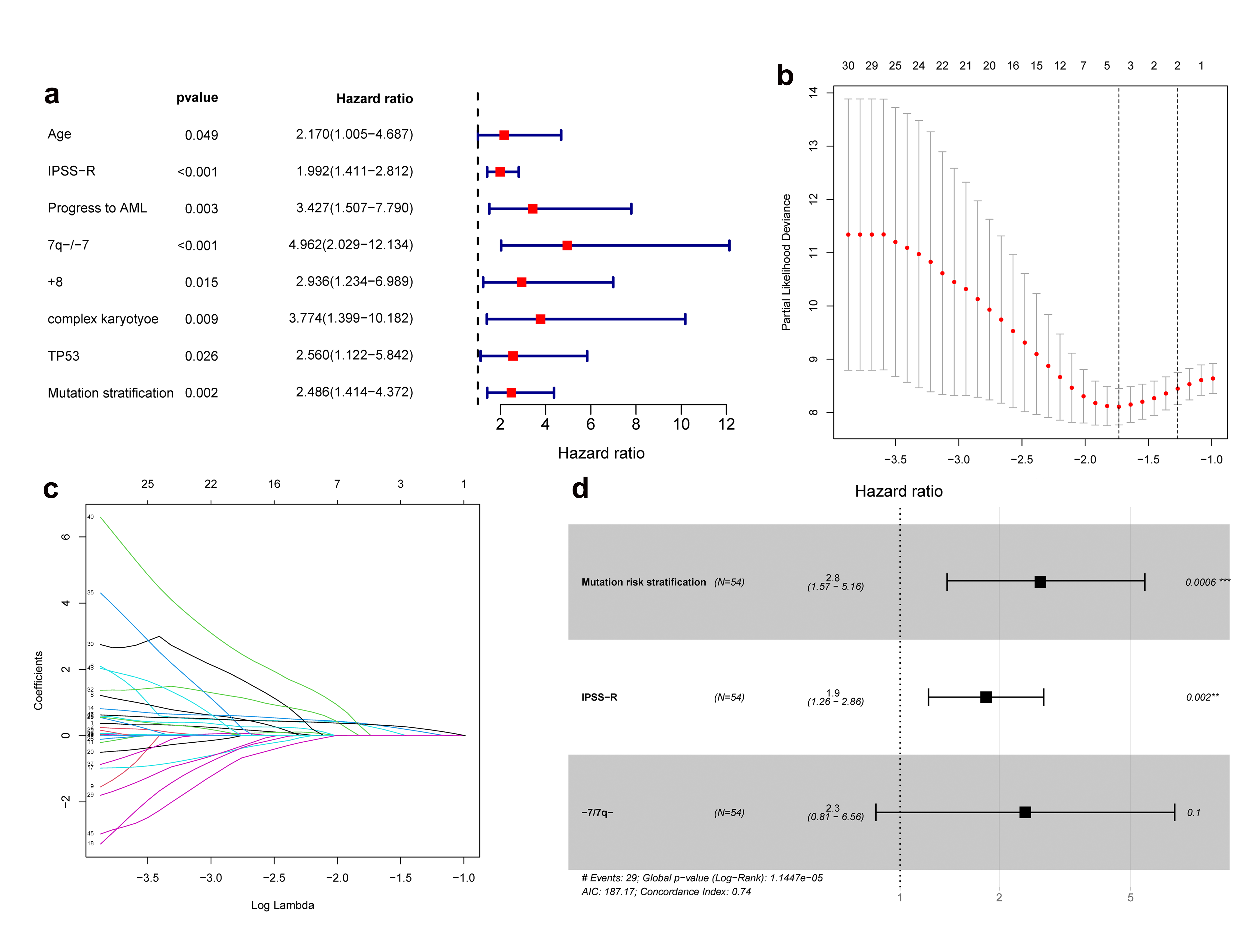

Supplement: Supplementary file 1 — Additional file 1. [file 12885_2021_7864_MOESM1_ESM.zip › supplementR4.png]
